# Generating colorful carrot germplasm through metabolic engineering of betalains pigments

**DOI:** 10.1093/hr/uhad024

**Published:** 2023-02-14

**Authors:** Yuan-Jie Deng, Ao-Qi Duan, Hui Liu, Ya-Hui Wang, Rong-Rong Zhang, Zhi-Sheng Xu, Ai-Sheng Xiong

**Affiliations:** National Key Laboratory of Crop Genetics and Germplasm Enhancement and Utilization, Ministry of Agriculture and Rural Affairs Key Laboratory of Biology and Germplasm Enhancement of Horticultural Crops in East China, College of Horticulture, Nanjing Agricultural University, 1 Weigang, Nanjing 210095, China; National Key Laboratory of Crop Genetics and Germplasm Enhancement and Utilization, Ministry of Agriculture and Rural Affairs Key Laboratory of Biology and Germplasm Enhancement of Horticultural Crops in East China, College of Horticulture, Nanjing Agricultural University, 1 Weigang, Nanjing 210095, China; National Key Laboratory of Crop Genetics and Germplasm Enhancement and Utilization, Ministry of Agriculture and Rural Affairs Key Laboratory of Biology and Germplasm Enhancement of Horticultural Crops in East China, College of Horticulture, Nanjing Agricultural University, 1 Weigang, Nanjing 210095, China; National Key Laboratory of Crop Genetics and Germplasm Enhancement and Utilization, Ministry of Agriculture and Rural Affairs Key Laboratory of Biology and Germplasm Enhancement of Horticultural Crops in East China, College of Horticulture, Nanjing Agricultural University, 1 Weigang, Nanjing 210095, China; National Key Laboratory of Crop Genetics and Germplasm Enhancement and Utilization, Ministry of Agriculture and Rural Affairs Key Laboratory of Biology and Germplasm Enhancement of Horticultural Crops in East China, College of Horticulture, Nanjing Agricultural University, 1 Weigang, Nanjing 210095, China; National Key Laboratory of Crop Genetics and Germplasm Enhancement and Utilization, Ministry of Agriculture and Rural Affairs Key Laboratory of Biology and Germplasm Enhancement of Horticultural Crops in East China, College of Horticulture, Nanjing Agricultural University, 1 Weigang, Nanjing 210095, China; National Key Laboratory of Crop Genetics and Germplasm Enhancement and Utilization, Ministry of Agriculture and Rural Affairs Key Laboratory of Biology and Germplasm Enhancement of Horticultural Crops in East China, College of Horticulture, Nanjing Agricultural University, 1 Weigang, Nanjing 210095, China

## Abstract

Betalains are tyrosine-derived plant pigments exclusively found in the Caryophyllales order and some higher fungi and generally classified into two groups: red-violet betacyanins and yellow-orange betaxanthins. Betalains attract great scientific and economic interest because of their relatively simple biosynthesis pathway, attractive colors and health-promoting properties. Co-expressing two core genes *BvCYP76AD1* and *BvDODA1* with or without a glycosyltransferase gene *MjcDOPA5GT* allowed the engineering of carrot (an important taproot vegetable) to produce a palette of unique colors. The highest total betalains content, 943.2 μg·g^−1^ DW, was obtained in carrot taproot transformed with p35S:RUBY which produces all of the necessary enzymes for betalains synthesis. Root-specific production of betalains slightly relieved tyrosine consumption revealing the possible bottleneck in betalains production. Furthermore, a unique volcano-like phenotype in carrot taproot cross-section was created by vascular cambium-specific production of betalains. The betalains-fortified carrot in this study is thus anticipated to be used as functional vegetable and colorful carrot germplasm in breeding to promote health.

## Introduction

Betalains, which are water-soluble natural pigments, are exclusively found in some plants of the Caryophyllales order and a small number of fungi [1, 2]. Betalains pigments are usually classified into two groups: the red-violet betacyanins group and yellow-orange betaxanthins group [3]. On account of their attractive colors and stability, betalains pigment is a good option for food colorants and natural dyes [4, 5]. Their powerful antioxidant activity and potential benefit for human health make betalains also a choice of therapeutic products [6–8]. Betalains have also been suggested as acting in attracting pollinators, stress defense, photoprotection mechanism, and so on [9–12].

**Figure 1:**
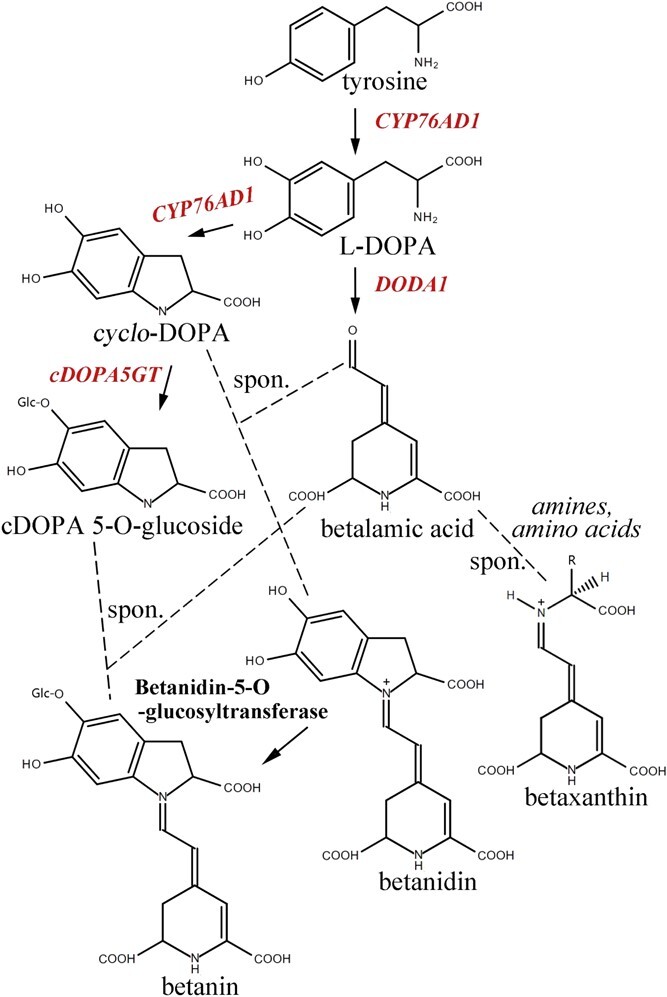
Representation of betalain biosynthetic pathway. Solid arrows show enzymatic reactions. Dotted arrows show spontaneous reactions. Genes used for betalain engineering in carrot are shown in red.

After decades of effort, the core biosynthesis pathway of betalains is successfully elucidated in beet (Fig. 1). First, tyrosine is hydroxylated by at least three enzymes (CYP76AD1, CYP76AD5, and CYP76AD6) to form 3,4-dihydroxy-L-phenyalanine (L-DOPA) redundantly [13, 14]. Then, DOPA 4,5-dioxygenase enzyme catalyzes the conversion of L-DOPA to betalamic acid, the core structure of betalains, in a two-step manner [15, 16]. Elsewise, L-DOPA is catalyzed with the help of aforementioned CYP76AD1 to form cyclo-DOPA [17, 18]. Betalamic acid condenses with cyclo-DOPA or amino acids/amines spontaneously to form the betacyanin precursor betanidin or betaxanthins, respectively [3, 17]. Finally, betanidin goes through glycosylation and acylation reactions yeilding multiple stable betacyanins, such as betanin and lampranthin I [3, 19]. However, a different pathway toward betanin occurs in some species where glycosylation happened before the condensation of betalamic acid with *cyclo*-DOPA [20].

Now is the golden age of betalains. So far, many efforts have been made to produce betalains in heterologous plant species through metabolic engineering, including tomato, rice, eggplant, tobacco, *Arabidopsis*, etc. [21–23]. Before the discovery of the first committed step, heterologous engineering of betalains depended on either substrate feeding or tyrosinase from mushroom [24, 25]. Because the precursor of betalains, tyrosine, exists in all plant species, it is feasible to produce betalains in almost every species through metabolic engineering. Based on this theory, a visual reporter, *RUBY*, containing all the key genes (*BvCYP76AD1*, *BvDODA1*, and *MjcDOPA5GT*) linked by 2A sequences was developed to monitor gene expression and plant transformation [22]. In addition to simply reconstructing the metabolic pathway, researchers aimed to pursue a higher production of betalains in the heterogenous system. A possible bottleneck of betalains production in red beets was proposed. It was found that tyrosine contents were positively correlated with betalain accumulation among red beets [26]. Typically, arogenate dehydrogenases involved in tyrosine synthesis were feedback-inhibited by tyrosine. However, BvADHα was reported to exhibit relaxed sensitivity to tyrosine [27]. Heterologous expression of *BvADHα* in *Nicotiana benthamiana* increased the tyrosine levels vastly [27]. Expression of *BvADHα* also improved the heterogenous production of betalains in *N. benthamiana*, lettuce, and tomato [28].

Carrot (*Daucus carota ssp. sativus*) is an important taproot vegetable worldwide providing rich numerous nutrients to humans [29]. For now, two groups of pigments, carotenoid and anthocyanin, are found in carrot taproots [30–33]. Here, we conducted metabolic engineering for a new group of pigment betalains in carrot utilizing the single open reading frame *RUBY* [22]. Carrots with different colors were generated *via* expression of *RUBY* and its short version *RUBY-S* without the *cDOPA5GT* gene*.* Root-specific and vascular cambium-specific production of betalains were generated by the use of *pDJ3S* and *p15* promoters, respectively. Our results improved understanding of heterogenous production of betalains in carrot and provided colorful germplasm for carrot breeding.

## Results

### Heterologous expression of *CYP76AD1* and *DODA1* is sufficient for betanin production in carrot

**Figure 2:**
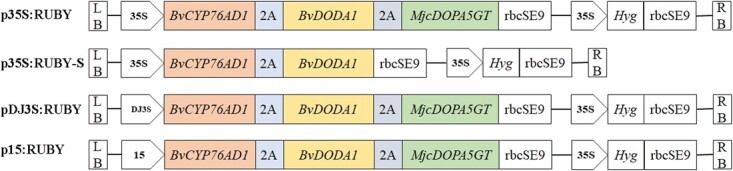
Schematic of the p35S:RUBY, p35S:RUBY-S, pDJ3S:RUBY, p15:RUBY vectors. *BvCYP76AD1*, *Beta vulgaris* cytochrome P450; *BvDODA1*, *B. vulgaris* DOPA 4,5-dioxygenase; *MjcDOPA5GT*, *Mirabilis jalapa* cyclo-DOPA-5-O-glucosyltransferase; *Hyg*, hygromycin resistance marker.

To produce betalains in carrot and better understand the biosynthesis pathway of betalains, two constructions were generated: p35S:RUBY and p35S:RUBY-S (Fig. 2). p35S:RUBY harboured *RUBY* driven by the CaMV 35S promoter. p35S:RUBY-S harboured a short version of *RUBY* (without *cDOPA5GT* gene) driven by the CaMV 35S promoter. These two constructions were transformed into yellow taproot carrot cultivar individually.

**Figure 3:**
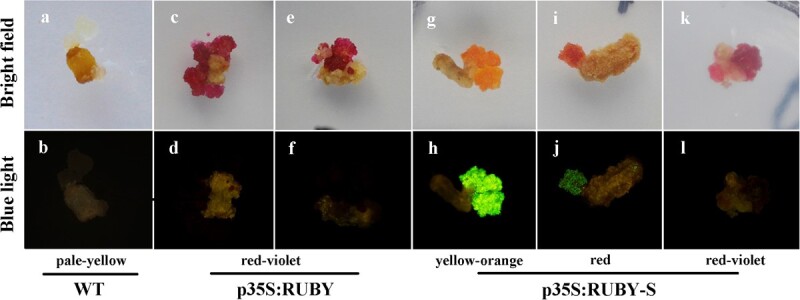
Phenotype of WT, p35S:RUBY, and p35S:RUBY-S callus under bright-field (top row) or blue light (bottom row). **a**–**b** callus induced from WT. **c**–**f** callus induced from p35S:RUBY. **g**–**l** callus induced from p35S:RUBY-S.

Callus transformed with p35S:RUBY turned red-violet, suggesting betacyanin production, while the wild type (WT) callus was pale yellow (Fig. 3a, c, and e). However, callus transformed with p35S:RUBY-S presented three phenotypes: yellow-orange, red, and red-violet (Fig. 3g, i, and k). Interestingly, the rate of yellow-orange callus was very low, less than 1%. Variation in p35S:RUBY-S callus color might result from the difference in betacyanin versus betaxanthin accumulation. In order to prove the hypothesis, carrot callus with different colors were then exposed to blue light in which betaxanthin had a typical green fluorescence. Bright green fluorescence was observed in yellow-orange callus (Fig. 3h). Green fluorescence in red callus was weaker than yellow-orange callus (Fig. 3h, j). No fluorescence was observed in red-violet callus and pale-yellow callus (Fig. 3l). These results proved the hypothesis that variation in p35S:RUBY-S callus color resulted from the differences in betacyanin versus betaxanthin accumulation.

**Figure 4:**
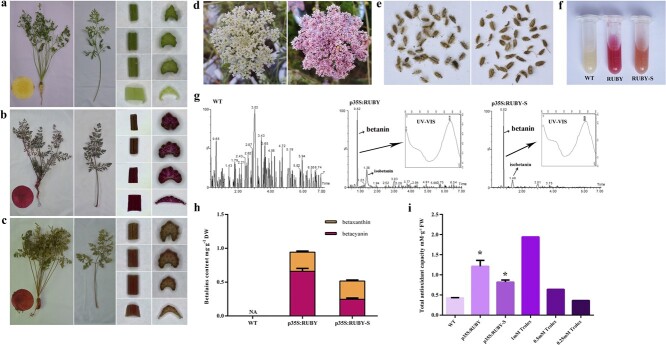
Engineering betalain production in carrot. **a** WT carrot plant phenotype including entire plant, taproot section, the entire petiole, four small cuts of petioles and their cross-sections sampling from the base to the top. **b** p35S:RUBY carrot plant phenotype including whole plant, taproot section, the entire petiole, four small cuts of petioles and their cross-sections sampling from the base to the top. **c** p35S:RUBY-S carrot plant phenotype including whole plant, taproot section, the entire petiole, four small cuts of petioles and their cross-sections sampling from the base to the top. **d** White flower from WT carrot (left) and betalains pigmented flowers from p35S:RUBY carrot (right). **e** Seeds of WT carrot (left) and p35S:RUBY carrot (right). **f** Colorization of betalains extract solution from WT (left), p35S:RUBY (mid), and p35S:RUBY-S (right) carrot taproots. **g** LC–MS extracted ion chromatogram of betanin/isobetanin corresponding mass (M + H = 551.1) and UV–VIS absorption of the betanin peak for WT, p35S:RUBY, and p35S:RUBY-S carrot taproots. **h** Betalains contents of WT, p35S:RUBY, and p35S:RUBY-S carrot taproots. Data are means of at least three biological replicates ± SDs. **i** Total antioxidant capacity of WT, p35S:RUBY, and p35S:RUBY-S carrot taproots. 0.25, 0.5, and 1 mM Trolox as positive controls. *Significant differences between transgenic carrots (p35S:RUBY, p35S:RUBY-S) and WT. *P < 0.05. Data represents means of at least three biological replicates ± SDs.

The taproot of WT carrot was yellow due to the minor accumulation of carotenoid (Fig. 4a). All overground parts of WT carrot were green. Entire carrot plants transformed with p35S:RUBY and p35S:RUBY-S both turned red, although p35S:RUBY transgenic plants were darker in color (Fig. 4b and c). Flowers of p35S:RUBY were red-violet compared with white flowers of WT carrot and p35S:RUBY transgenic plants could produce viable seeds (Fig. 4d and e). Seed color of p35S:RUBY was the same as WT. Because p35S:RUBY-S transgenic plants missed the season of vernalization, we didn’t obtain the flower of p35S: RUBY-S. It is a noteworthy fact that p35S:RUBY transgenic carrot taproot was red-violet compared with red carrot taproot transformed with p35S:RUBY-S. Also, extraction solutions of p35S:RUBY and p35S:RUBY-S taproots were red-violet and orange-red, respectively (Fig. 4f).

The extraction solution of WT taproot was pale yellow. Liquid chromatography-mass spectrometry (LC–MS) analysis of carrot taproots derived from p35S:RUBY and p35S:RUBY-S transgenic plants confirmed that betanin and isobetanin were both present in these two transformants (Fig. 4g). Betalain contents in taproots of p35S:RUBY and p35S:RUBY-S transgenic plants were also measured (Fig. 4h). Taproots of p35S:RUBY transgenic plants accumulated 660.3 μg·g^−1^ DW betacyanin and 282.9 μg·g^−1^ DW betaxanthin. Taproots of p35S:RUBY-S transgenic plants accumulated 246.9 μg·g^−1^ DW betacyanin and 269.48 μg·g^−1^ DW betaxanthin. Difference in betalains content and ratio of betacyanin/betaxanthin explained the color variation between p35S:RUBY and p35S:RUBY-S taproots. Despite the presence of yellow-orange callus transformed with p35S:RUBY-S vector, no yellow-orange group carrots were obtained due to the low probability of yellow-orange callus and relative low regeneration efficiency of carrots. These results together suggested that heterologous expression of *CYP76AD1* and *DODA1* was sufficient for betanin production in carrot.

Betalains were reported to possess strong antioxidant capacity [8, 34]. Hence, total antioxidant capacity in taproots of p35S:RUBY, p35S:RUBY-S, and WT was measured using ferric reducing ability of plasma (FRAP) assay (Fig. 4i). As expected, total antioxidant capacity of p35S:RUBY and p35S:RUBY-S taproots were both higher than that of WT and 0.5 mM Trolox (positive control). Total antioxidant capacity of p35S:RUBY taproots were the strongest and were 2.8 times as much as WT. We obtained eight transgenic carrots hosting p35S:RUBY and they all showed higher antioxidant capacity than p35S:RUBY-S. Total antioxidant capacity of p35S:RUBY-S taproots was 1.9 times as much as WT. The different total antioxidant capacity between p35S:RUBY and p35S:RUBY-S might have resulted from the levels of betalain contents.

### Root-specific production of betalains in carrot

**Figure 5:**
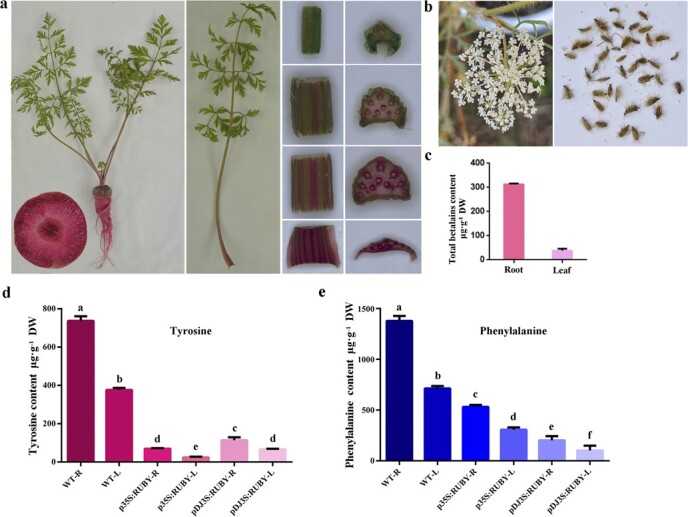
Root-specific production of betalains. **a** pDJ3S:RUBY carrot plant phenotype including whole plant, taproot section, the entire petiole, four small cuts of petioles and their cross-sections, sampling from the base to the top. **b** White flower and seeds from pDJ3S:RUBY carrot. **c** Total betalain contents of taproots and leaves of pDJ3S:RUBY carrot. Data are means of at least three biological replicates ± SDs. **d** Tyrosine contents of taproots and leaves from WT, p35S:RUBY, and pDJ3S:RUBY plants. Data represents means of at least three biological replicates ± SDs. **e** Phenylalanine contents of taproots and leaves from WT, p35S:RUBY, and pDJ3S:RUBY plants. Values are means of at least three biological replicates ± SDs.

In order to restrict the production of betalains to taproot and test the potential of avoiding a dramatic decrease of tyrosine level by a root-specific promoter, we determined to drive the *RUBY* in a root-specific manner. Previously, a promoter fragment (*pDJ3S*) isolated from *Dioscorea japonica* was proved to drive preferential and high expression level of genes in carrot taproot [35, 36]. Therefore, root-specific production of betalains may be feasible. To this end, we replaced the CaMV 35S promoter of p35S:RUBY with the *pDJ3S* promoter, resulting in pDJ3S:RUBY (Fig. 2). At the callus stage, we observed strong expression of *RUBY* depending on red-violet color (Fig. S1, see online supplementary material). Four-month-old transgenic carrot plants were harvested for phenotype analysis (Fig. 5a). Indeed, a preferential and high expression level of *RUBY* was obtained in carrot roots. Meanwhile, obvious expression was observed at the base of the petiole and expression decreased gradually from the base of the petiole to the top. The leaf blade stayed green, indicating no obvious expression of *RUBY*. Also, no obvious betalain accumulation was observed in flowers and the transformed plants could produce viable seeds (Fig. 5b). Total betalain contents of taproots and leaves derived from pDJ3S:RUBY were determined and taproots accumulated 313.1 μg·g^−1^ DW betalains, 8.8 times as much as leaves (Fig. 5c).

Tyrosine contents of WT, p35S:RUBY, and pDJ3S:RUBY were determined and compared (Fig. 5d). Leaves and taproots of WT accumulated 376.5 and 737.1 μg·g^−1^ DW tyrosine, respectively. Tyrosine contents decreased dramatically in leaves and taproots of p35S:RUBY and pDJ3S:RUBY carrots compared with WT. Leaves and taproots of p35S:RUBY carrots accumulated 24.1 and 69.4 μg·g^−1^ DW tyrosine, respectively. Leaves and taproots of pDJ3S:RUBY carrots accumulated 66.5 and 113.4 μg·g^−1^ DW tyrosine, respectively. Compared with WT, tyrosine contents decreased about 15.6-fold and 10.6-fold in leaves and taproots of p35S:RUBY carrots, respectively. Indeed, replacing the CaMV 35S promoter of p35S:RUBY with *pDJ3S* promoter mildly alleviated the excessive consumption of tyrosine. The leaves and taproots tyrosine contents of pDJ3S:RUBY were 1.6 and 2.8 times as much as that of p35S:RUBY, respectively.

Phenylalanine contents were also checked because of the competitional relationship between tyrosine and phenylalanine [26]. An obvious decline of phenylalanine contents was observed (Fig. 5e). The phenylalanine contents of leaves and taproots of p35S:RUBY decreased 2.3-fold and 2.6-fold compared with WT. Ten transgenic carrots hosting pDJ3S:RUBY-S were obtained and they all showed a lower amount of phenyalanine compared to p35S:RUBY carrots. The phenylalanine contents of leaves and taproots of pDJ3S:RUBY decreased 7.0-fold and 6.8-fold compared with WT. These results demonstrated the root-specific production of betalains by the use of *pDJ3S* promoter and huge consumption of tyrosine for betalain production even in a root-specific manner.

### Vascular cambium specific production of betalains resulting in volcano-like phenotype in taproot cross-sections

Inspired by the purple peridermal carrot phenotype (carrot accumulating anthocyanins exclusively in taproot periderm), it would be of interest to ‘color’ carrot with betalains at a particular part of the taproot cross-section [31, 32, 37]. It has been reported that the cassava *p15* promoter led to very intense GUS staining of the vascular cambium in carrot taproots [35, 38]. Therefore, it may be feasible to restrict the production of betalains to the vascular cambium of carrot taproot, thus creating an attractive carrot phenotype with a red circle in the middle of the cross-section.

**Figure 6:**
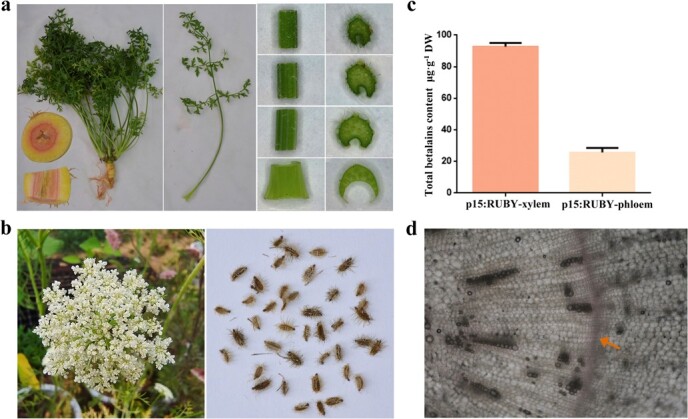
Vascular cambium-specific production of betalains in taproot cross-section. **a** Phenotype of p15:RUBY carrot plant including whole plant, taproot cross-section, taproot vertical section, the entire petiole, four small cuts of petioles and their cross-sections, sampling from the base to the top. **b** White flower and seeds from p15:RUBY carrot. **c** Total betalain contents in xylem and phloem of carrot taproots. Data represents means of at least three biological replicates ± SDs. **d** Observation of taproot cross-section of p15:RUBY transgenic carrot in bright-field microscopy. Orange arrow indicated the vascular cambium. Xylem and phloem were on the left and right of vascular cambium, respectively.

To this end, we replaced the CaMV 35S promoter of p35S:RUBY by *p15* promoter resulting in p15:RUBY (Fig. 2). p15:RUBY was transformed into the pale yellow carrot cultivar ‘QTH’ in which the red-violet color of betalains is obvious. At the callus stage, minor expression of *RUBY* was observed depending on pale red color (Fig. S2, see online supplementary material). When carrots were four months old and taproots expanded, carrot plants were harvested for phenotype analysis (Fig. 6a). The lateral roots and incompletely expanded parts of taproots periderm were pale red, and the fully expanded parts of taproot were pale yellow, the same as WT plants. The vascular cambium was intense red in the middle of the cross-section due to the high accumulation of betalains. In a difference from the previous report that *p15* promoter led to exclusive expression in vascular cambium, xylem also appeared pale red, suggesting a minor expression of *RUBY*. Phloem showed no obvious color change, indicating little betalain accumulation. No obvious betalain accumulation was observed in flowers (Fig. 6b). p15:RUBY transgenic plants could produce viable seeds (Fig. 6b). Total betalain content of xylem,92.7 μg·g^−1^ DW including vascular cambium was 3.6 times as much as phloem, 25.8 μg·g^−1^ DW including periderm (Fig. 6c). In bright-field microscopy, a red coloration was observed in the vascular cambium cells of the cross-section (Fig. 6d). These cells were tightly packed and arranged in a ring. Thus, an attractive carrot phenotype with a red circle in the middle of the cross-section was created by the use of p15:RUBY.

## Discussion

Betalains are highly attractive pigments found in the Caryophyllales order and some higher fungi [1, 39]. In addition, the relatively simple biosynthesis pathway has attracted the attention of many researchers [22]. Here, we demonstrated that heterologous engineering of betalains can be achieved in carrot, an important taproot vegetable around the world. The role of cyclo-DOPA-5-O-glucosyltransferase involved in the betalains biosynthesis in carrot was revealed. Although *MjcDOPA5GT* was not necessary for betanin production in carrot, it could drive metabolism towards betanin and increase the betalain contents. Heterologous expression of *BvCYP76AD1* and *BvDODA1* caused the disorder of betalains metabolism in callus, resulting in three phenotypes (orange, red, and red-violet). The difference of the three phenotypes resulted from the betacyanin/betaxanthin ratio. This was similar to the results that red and yellow types of rice were obtained by the introduction of three genes (*melo*, *BvCYP76AD1*, *BvDODA1*), although *melo* and *BvCYP76AD1* were redundant [23]. It is reported that the ratio of betacyanin/betaxanthin production can be manipulated by expression of *CYP76AD1*, *CYP76AD6*, or a combination of both genes [21]. In this study, we demonstrated that the ratio of betacyanin/betaxanthin production could be similarly manipulated by the expression of *cDOPA5GT*. Transient expression of *BvCYP76AD1* and *BvDODA1* resulted in the production of a metabolite with the same mass as betanin [28]. Also, an unidentified peak with a UV spectrum characteristic of betaxanthins was detected [28]. These findings and our results proved the involvement of endogenous enzymes with a similar function as glucosyltransferase for betalains biosynthesis in many species. However, expression of two genes, *CYP76AD1* and *DODA*, in *Arabidopsis* did not produce betalains in the absence of tyrosine feeding [14]. In another work, *Arabidopsis* transformed with *RUBY* containing all the key genes (*CYP76AD1*, *DODA1*, and *cDOPA5GT*) turned red in the whole plants, suggesting that there was no similar endogenous glucosyltransferase, or substrate supply was far from sufficient in *Arabidopsis* [22]. Except for the aforementioned glucosylation, unexpected betanin modification (e.g. acylation and glucosylation) was reported following the biofortification of three vegetables with betalains [21]. After decades of effort in elucidating the core biosynthesis pathway of betalains, the modification mechanism remains to be further studied.

The precursor of betalains, tyrosine, is an important primary metabolic product required for protein biosynthesis [26]. It serves as a precursor of many plant natural products, such as tocopherol, rosmarinic acid, dhurrin, and plastoquinone [40–43]. More and more studies had shown that the high content of betalains in red beets was related to the tyrosine supply mechanism [26, 27]. The relationship between betalains and tyrosine content has not been studied in heterogenous betalain production experiments. We investigated the bottleneck of betalain biosynthesis in carrots by comparing tyrosine contents in WT, p35S:RUBY, and pDJ3S:RUBY. A dramatic decrease of tyrosine contents in p35S:RUBY and pDJ3S:RUBY carrots was observed, although root-specific production of betalains showed relatively less decrease than p35S:RUBY. Moreover, the contents of phenylalanine competing carbon flow with tyrosine also decreased greatly [27]. The phenylalanine contents of pDJ3S:RUBY were lower than p35S:RUBY and showed no strict correlation with the betalain contents, suggesting indirect effect of betalain production on phenylalanine metabolism. These results demonstrated that production of betalains consumed the substrate greatly and might drive the carbon flow from the phenylalanine pathway to the tyrosine pathway. Although betalain biosynthesis affected the metabolism of plant cells by the reduction of tyrosine and phenylalanine, all the transformed carrot plants could produce viable seeds. Seed color of p35S:RUBY was the same as WT, which might be due to the degradation of betalains in fully mature seeds. For further improving betalain production in carrot, future attention should be paid to the supply of tyrosine. Recently, BvADHa was found to exhibit relaxed sensitivity to tyrosine and overexpression of this enzyme enhanced tyrosine content in tobacco leaves [27]. Simultaneous overexpression of this gene together with core biosynthesis genes in tomato and rice did increase betalain production [28, 44]. Therefore, this enzyme may also be a critical tool to enhance the heterogenous production of betalains in carrots.

Although carrot taproots accumulate large amount of pigments, only a very limited variety of pigments are found. Natural carrots are mainly divided into carotenoid and anthocyanin groups [32, 45, 46]. Carotenoid group carrots show great variations in taproot color, white, yellow, orange, and red, depending on the types and amounts of carotenoids [33, 47–49]. The taproots of anthocyanin group carrots containing anthocyanins are always purple [50, 51]. There are many variations of this kind of carrot, ranging from the purple peridermal carrot type (purple periderm but nonpurple phloem and xylem) to the solid purple carrot type (purple periderm, phloem, and xylem) [32, 51]. In this study, a new pigment category of carrots (betalain group) was created through metabolic engineering, showing a unique red to red-violet color. Under the influence of tissue culture and genetic transformation, the fleshy root of transgenic carrot exhibited a certain degree of torsion [31, 33, 52]. No obvious unfavorable effect of betalain biosynthesis on carrot root size and yield was observed, which would be important if these betalain group carrots are used for production. Inspired by extensive variations of anthocyanin group carrots, we sought to create new type of carrot variants with betalains. The *p15* promoter was used to drive *RUBY* expression in vascular cambium of taproot cross-section. Specific high expression was achieved in vascular cambium as expected. However, minor expression was achieved in xylem, which was different from previous reports, possibly due to the insensitivity of GUS staining or mutations in the *p15* promoter sequence [35]. p15:RUBY carrot taproots showed a bright red ring pigmented by betalains, like an erupting volcano, in the middle of taproot cross-sections. This type of pigmentation was not found in natural carrots, even the variant-rich anthocyanin group carrot. Therefore, this type of carrot may have the potential to be used as a breeding material and for commercial exploration. In addition, it is interesting to identify new characteristic promoters, such as epidermal specific promoters, phloem specific promoters, and xylem specific promoters. By combining three main pigments (betalains, carotenoids, and anthocyanins) and different promoters, a series of attractive carrot phenotypes can be created.

## Materials and methods

### Plant materials and growth conditions

The pale-yellow carrot cultivar, namely ‘Qitouhuang, QTH’, was selected for the experiment. All the carrots were grown in artificial climate room as previously described by Li *et al.* [33, 53].

### Vector construction

The single open reading frame *RUBY* contained three key genes, *BvCYP76AD1*, *BvDODA1*, and *MjcDOPA5GT*, which were linked by 2A peptides sequences. It was amplified from *pDR5:RUBY* [22] and inserted into the pCAMBIA 1301 vector between the CaMV 35S promoter and the pea (*Pisum sativum*) rbcSE9 terminator to create p35S:RUBY*.* The short version of *RUBY* (without *cDOPA5GT*) was also amplified to create p35S:RUBY-S*.* The *p15* promoter was amplified from cassava and *pDJ3S* promoter was chemically synthesized. The CaMV 35S promoter of p35S:RUBY was replaced with *pDJ3S* and *p15* promoters resulting in pDJ3S:RUBY and p15:RUBY, respectively. *RUBY*, *RYBU-S*, *p15*, and *pDJ3S* sequences are provided in Appendix S1 (see online supplementary material).

### Generation of transgenic carrot

All the constructs were introduced into *Agrobacterium tumefaciens* (GV3101) and transformed into carrots according to a method previously described [31, 32]. Four-month-old WT and transgenic carrots were harvested. Entire plant, taproot section, an intact petiole, four small cuts of petioles from the base to the top, and their cross-sections were sampled and shotted.

### Light and fluorescence microscopy

Freehand slicing of carrot taproot cross-section was made using a sharp knife for light microscopy observation. Nikon ECLIPSE E100 light microscope (Tokyo, Japan) equipped with a 20× objective was used for the visualization of betalains in the vascular cambium of carrot taproot. The fluorescence under blue light was observed using a fluorescent microscope (Olympus MVX10, Tokyo, Japan). Accumulation of betalains in petiole cross-sections of carrots was observed using a three-dimensional microscope (Leica DVM6A, Weztlar, Germany).

### Betalain extraction and measurement

Total betalains was extracted and quantitatively analysed according to the previously described method [21]. Betanin and isobetanin was identified by LC–MS system. Samples were separated on a reverse phase UPLC BEH column (100 × 2.1 mm, 1.7 μm; Waters Acquity) using a three-stage LC program. The gradient was set as the following (%A/%B): 95/5 for 0–0.5 min; 0.5–11.5 min, linear elution to 5/95; and 5/95 for 11.5–13.5 min. The mobile phase was as follows: 0.1% formic acid in water (A) and 0.1% formic acid in acetonitrile (B). Mass spectrometry detection was performed in positive ion mode with a selected m/z ranging from 100 to 1200. Other parameters were set as following: capillary, 2.5 kV; sample cone, 40 V. Spectral data (260–600 nm) were collected using a PDA detector.

### Determination of tyrosine and phenylalanine content

For tyrosine and phenylalanine extraction, 1 mL of 80% ethanol was added to 150 mg of dry powder sample in a mortar and the sample was ground until ethanol volatilized completely. The mortar was washed with 3 mL of 4% sulfosalicylic acid into a 10 mL centrifuge tube. Tyrosine and phenylalanine were extracted by ultrasonic vibration for 20 min. After centrifugation at 4000 rpm for 10 min, the supernatant was transferred to another 10 mL centrifuge tube and the extraction repeated twice. The supernatant was filtered with a 0.22 mm filter. Tyrosine and phenylalanine content were quantitatively analysed using an automatic amino acid analyzer (Hitachi, L-8900, Japan). Tyrosine and phenylalanine were monitored using a dual-channel UV detector at 570 nm. Tyrosine and phenylalanine concentration of samples were quantified according to their respective standard curves.

### Total antioxidant capacity assay

Total antioxidant capacity of carrot taproots was estimated using a commercialized kit (Beyotime, China) with the FRAP method following the manufacturer’s instructions. Total antioxidant capacity was indicated as concentration equivalents of FeSO4 standard solution.

### Statistical analysis

Data were statistically processed using SPSS 20.0 software, and the statistical difference of tyrosine and phenylalanine content between samples was determined using Duncan’s method at *P* < 0.05.

## Supplementary Material

Web_Material_uhad024

## Data Availability

The authors confirm that the data supporting the findings of this study are available within the article and its supplementary materials.
