# Supplementary material for: Generating colorful carrot germplasm through metabolic engineering of betalains pigments
Source: Hortic Res. 2023 Feb 14;10(4):uhad024. doi: 10.1093/hr/uhad024 (PMC10541523; doi:10.1093/hr/uhad024)

**Appendix S1:**

**The sequence information of *RUBY*, *RYBU-S*, *p15* and *pDJ3S* as follow:**

***RUBY:***

ATGGATCATGCGACCCTCGCCATGATCCTCGCGATCTGGTTCATCAGCTTCCACTTCATCAAGCTGCTGTTCTCCCAGCAGACCACCAAGCTGCTTCCGCCAGGACCAAAGCCGCTTCCGATCATCGGCAACATCCTTGAGGTGGGCAAGAAGCCGCATCGGTCCTTCGCCAACCTCGCCAAGATTCACGGCCCACTCATTTCCCTCAGACTCGGCTCTGTGACCACCATCGTTGTGTCCTCTGCCGACGTGGCCAAAGAGATGTTCCTCAAGAAGGATCACCCGCTCTCCAACCGCACGATCCCGAATAGTGTTACAGCCGGCGACCACCACAAGCTCACCATGTCTTGGCTCCCGGTGTCTCCGAAGTGGCGCAACTTCCGCAAGATTACCGCCGTGCATCTGCTCTCCCCACAGAGACTCGATGCCTGCCAGACATTCAGGCACGCCAAGGTGCAGCAGCTCTACGAGTACGTTCAAGAGTGCGCCCAGAAAGGCCAGGCCGTGGATATTGGCAAGGCCGCCTTTACGACCAGCCTCAACCTCCTCAGCAAGCTGTTCTTCAGCGTCGAGCTGGCGCACCACAAGTCCCATACCAGCCAAGAGTTCAAAGAGCTGATCTGGAACATCATGGAAGATATAGGCAAGCCGAACTACGCCGACTACTTCCCGATTCTCGGCTGCGTTGACCCATCTGGCATTAGAAGAAGGCTCGCCTGCTCCTTCGACAAGCTGATCGCCGTGTTCCAGGGCATCATCTGCGAGAGACTCGCCCCAGATTCCTCCACCACAACTACCACCACCACCGACGACGTGCTCGATGTGCTCCTCCAGCTGTTCAAGCAGAACGAGCTGACGATGGGCGAGATCAACCACCTCCTCGTGGACATCTTCGACGCCGGCACCGATACCACATCCTCCACATTCGAGTGGGTGATGACCGAGCTGATCCGCAATCCAGAGATGATGGAAAAGGCCCAAGAGGAAATCAAGCAGGTCCTCGGCAAGGACAAGCAGATCCAAGAGTCCGACATCATCAACCTGCCGTACCTCCAGGCGATCATCAAAGAGACACTCCGCCTCCATCCGCCGACCGTGTTCTTGCTCCCAAGAAAGGCCGACACCGATGTCGAGCTGTACGGCTACATCGTGCCGAAGGATGCCCAGATCCTCGTGAACCTCTGGGCCATTGGCAGGGACCCAAACGCCTGGCAGAACGCCGATATTTTCAGCCCAGAGCGCTTCATCGGCTGCGAGATCGATGTTAAGGGCCGCGATTTCGGCCTCCTTCCATTTGGCGCTGGCCGCAGAATTTGCCCAGGCATGAATCTCGCCATCAGGATGCTCACCCTCATGCTCGCCACACTCCTCCAGTTCTTCAACTGGAAGCTCGAAGGCGACATCTCCCCGAAGGACCTCGACATGGACGAGAAGTTCGGCATTGCGCTCCAAAAGACCAAGCCGCTCAAGCTCATCCCGATTCCGCGCTACCAATTGTTGAATTTTGATTTGTTGAAGTTGGCTGGAGATGTTGAATCTAATCCTGGACCTAAGATGATGAACGGCGAGGACGCCAACGACCAGATGATCAAAGAGTCCTTCTTCATCACCCACGGCAACCCGATCCTCACCGTCGAGGATACACATCCGCTCAGGCCGTTCTTCGAGACATGGCGCGAGAAGATTTTCTCCAAGAAGCCGAAGGCCATCCTCATCATCTCCGGCCACTGGGAGACAGTGAAGCCAACCGTGAACGCCGTGCACATCAACGACACCATCCACGACTTCGACGACTACCCAGCCGCCATGTACCAGTTCAAGTACCCAGCTCCAGGCGAGCCAGAGCTTGCGAGAAAGGTGGAAGAGATCCTCAAGAAGTCCGGGTTCGAGACAGCCGAGACAGACCAAAAGAGGGGCCTTGATCACGGCGCCTGGGTTCCACTCATGCTCATGTATCCAGAGGCGGACATCCCGGTGTGCCAGCTCTCAGTTCAGCCACATCTCGACGGCACCTACCACTACAATCTCGGCAGAGCCCTCGCGCCGCTCAAGAATGATGGCGTGCTCATTATTGGCTCCGGCAGCGCCACACATCCACTCGATGAGACACCGCACTACTTCGATGGTGTTGCCCCTTGGGCCGCTGCCTTCGATTCTTGGCTTAGGAAGGCCCTCATCAACGGCCGCTTCGAGGAAGTGAACATCTACGAGAGCAAGGCCCCGAACTGGAAGCTCGCCCATCCATTTCCAGAGCACTTCTACCCGCTCCACGTTGTGCTCGGCGCTGCTGGTGAAAAGTGGAAGGCCGAGCTGATCCACTCCTCCTGGGATCATGGCACACTTTGCCACGGCTCCTACAAGTTCACCTCCGCCCAATTGTTGAATTTTGATTTGTTGAAGTTGGCTGGAGATGTTGAATCTAATCCTGGACCTACCGCCATCAAGATGAACACCAACGGCGAGGGCGAGACACAGCACATCCTCATGATCCCGTTCATGGCGCAGGGCCACCTCAGGCCATTTCTCGAACTCGCCATGTTCCTCTACAAGCGCTCCCACGTGATCATCACCCTGCTCACAACTCCGCTCAACGCCGGCTTCCTCAGGCACCTCCTTCACCACCATTCCTACTCCTCCAGCGGCATCAGGATCGTCGAGCTGCCATTCAACTCCACCAACCACGGACTCCCACCGGGCATCGAGAACACCGATAAGCTCACACTCCCGCTCGTGGTGTCCCTCTTCCATTCCACCATCAGCCTCGATCCGCACCTCCGCGATTACATCTCCAGGCATTTCAGCCCAGCCAGGCCACCACTCTGCGTGATCCATGATGTGTTCCTCGGCTGGGTTGACCAGGTGGCCAAGGATGTGGGCTCTACAGGCGTGGTGTTCACAACAGGCGGCGCTTATGGCACATCCGCCTACGTGTCCATCTGGAACGATCTCCCGCACCAGAACTACTCCGACGACCAAGAGTTCCCGCTGCCAGGCTTCCCAGAGAACCATAAGTTCCGCAGGTCCCAGCTCCATCGGTTCCTCAGATATGCCGACGGCTCCGACGATTGGTCCAAGTATTTCCAGCCGCAGCTCCGCCAGTCCATGAAGTCTTTTGGCTGGCTCTGCAACTCCGTGGAAGAGATCGAGACACTCGGCTTCTCCATCCTCCGCAACTACACCAAGCTGCCGATCTGGGGCATCGGCCCACTTATTGCTTCCCCAGTGCAGCACTCCTCCTCCGACAACAATTCAACAGGCGCCGAGTTCGTGCAGTGGCTCAGCCTCAAAGAGCCGGACTCCGTCCTCTACATCTCCTTCGGCTCCCAGAACACGATCAGCCCGACGCAGATGATGGAACTCGCTGCTGGCCTTGAGTCCTCCGAGAAGCCATTCCTCTGGGTGATCAGAGCCCCGTTCGGCTTCGACATCAACGAAGAGATGCGCCCAGAGTGGCTGCCAGAGGGCTTTGAGGAACGCATGAAGGTGAAGAAACAGGGCAAGCTCGTGTACAAGCTCGGCCCGCAGCTTGAGATCCTCAACCATGAATCCATCGGCGGCTTTCTCACCCACTGCGGATGGAACAGCATCCTTGAGTCTCTTCGCGAGGGCGTTCCGATGCTTGGATGGCCACTTGCTGCCGAGCAGGCCTACAACCTCAAGTACCTCGAAGATGAGATGGGCGTCGCGGTTGAGCTTGCTAGAGGCCTCGAAGGCGAGATCTCCAAAGAGAAGGTCAAGCGCATCGTCGAGATGATCCTTGAGCGCAACGAGGGCTCCAAAGGCTGGGAGATGAAGAATCGCGCCGTGGAAATGGGCAAAAAGCTCAAGGACGCCGTGAACGAGGAAAAAGAGCTGAAGGGCTCCTCCGTGAAGGCGATCGACGATTTCCTCGACGCCGTCATGCAGGCCAAACTTGAGCCAAGCCTCCAGTGA

***RUBY-S:***

ATGGATCATGCGACCCTCGCCATGATCCTCGCGATCTGGTTCATCAGCTTCCACTTCATCAAGCTGCTGTTCTCCCAGCAGACCACCAAGCTGCTTCCGCCAGGACCAAAGCCGCTTCCGATCATCGGCAACATCCTTGAGGTGGGCAAGAAGCCGCATCGGTCCTTCGCCAACCTCGCCAAGATTCACGGCCCACTCATTTCCCTCAGACTCGGCTCTGTGACCACCATCGTTGTGTCCTCTGCCGACGTGGCCAAAGAGATGTTCCTCAAGAAGGATCACCCGCTCTCCAACCGCACGATCCCGAATAGTGTTACAGCCGGCGACCACCACAAGCTCACCATGTCTTGGCTCCCGGTGTCTCCGAAGTGGCGCAACTTCCGCAAGATTACCGCCGTGCATCTGCTCTCCCCACAGAGACTCGATGCCTGCCAGACATTCAGGCACGCCAAGGTGCAGCAGCTCTACGAGTACGTTCAAGAGTGCGCCCAGAAAGGCCAGGCCGTGGATATTGGCAAGGCCGCCTTTACGACCAGCCTCAACCTCCTCAGCAAGCTGTTCTTCAGCGTCGAGCTGGCGCACCACAAGTCCCATACCAGCCAAGAGTTCAAAGAGCTGATCTGGAACATCATGGAAGATATAGGCAAGCCGAACTACGCCGACTACTTCCCGATTCTCGGCTGCGTTGACCCATCTGGCATTAGAAGAAGGCTCGCCTGCTCCTTCGACAAGCTGATCGCCGTGTTCCAGGGCATCATCTGCGAGAGACTCGCCCCAGATTCCTCCACCACAACTACCACCACCACCGACGACGTGCTCGATGTGCTCCTCCAGCTGTTCAAGCAGAACGAGCTGACGATGGGCGAGATCAACCACCTCCTCGTGGACATCTTCGACGCCGGCACCGATACCACATCCTCCACATTCGAGTGGGTGATGACCGAGCTGATCCGCAATCCAGAGATGATGGAAAAGGCCCAAGAGGAAATCAAGCAGGTCCTCGGCAAGGACAAGCAGATCCAAGAGTCCGACATCATCAACCTGCCGTACCTCCAGGCGATCATCAAAGAGACACTCCGCCTCCATCCGCCGACCGTGTTCTTGCTCCCAAGAAAGGCCGACACCGATGTCGAGCTGTACGGCTACATCGTGCCGAAGGATGCCCAGATCCTCGTGAACCTCTGGGCCATTGGCAGGGACCCAAACGCCTGGCAGAACGCCGATATTTTCAGCCCAGAGCGCTTCATCGGCTGCGAGATCGATGTTAAGGGCCGCGATTTCGGCCTCCTTCCATTTGGCGCTGGCCGCAGAATTTGCCCAGGCATGAATCTCGCCATCAGGATGCTCACCCTCATGCTCGCCACACTCCTCCAGTTCTTCAACTGGAAGCTCGAAGGCGACATCTCCCCGAAGGACCTCGACATGGACGAGAAGTTCGGCATTGCGCTCCAAAAGACCAAGCCGCTCAAGCTCATCCCGATTCCGCGCTACCAATTGTTGAATTTTGATTTGTTGAAGTTGGCTGGAGATGTTGAATCTAATCCTGGACCTAAGATGATGAACGGCGAGGACGCCAACGACCAGATGATCAAAGAGTCCTTCTTCATCACCCACGGCAACCCGATCCTCACCGTCGAGGATACACATCCGCTCAGGCCGTTCTTCGAGACATGGCGCGAGAAGATTTTCTCCAAGAAGCCGAAGGCCATCCTCATCATCTCCGGCCACTGGGAGACAGTGAAGCCAACCGTGAACGCCGTGCACATCAACGACACCATCCACGACTTCGACGACTACCCAGCCGCCATGTACCAGTTCAAGTACCCAGCTCCAGGCGAGCCAGAGCTTGCGAGAAAGGTGGAAGAGATCCTCAAGAAGTCCGGGTTCGAGACAGCCGAGACAGACCAAAAGAGGGGCCTTGATCACGGCGCCTGGGTTCCACTCATGCTCATGTATCCAGAGGCGGACATCCCGGTGTGCCAGCTCTCAGTTCAGCCACATCTCGACGGCACCTACCACTACAATCTCGGCAGAGCCCTCGCGCCGCTCAAGAATGATGGCGTGCTCATTATTGGCTCCGGCAGCGCCACACATCCACTCGATGAGACACCGCACTACTTCGATGGTGTTGCCCCTTGGGCCGCTGCCTTCGATTCTTGGCTTAGGAAGGCCCTCATCAACGGCCGCTTCGAGGAAGTGAACATCTACGAGAGCAAGGCCCCGAACTGGAAGCTCGCCCATCCATTTCCAGAGCACTTCTACCCGCTCCACGTTGTGCTCGGCGCTGCTGGTGAAAAGTGGAAGGCCGAGCTGATCCACTCCTCCTGGGATCATGGCACACTTTGCCACGGCTCCTACAAGTTCACCTCCGCCTAA

***p15:*** AAATTATATTAAATTTATTTATAATATAATCGAGTTTAAATTTGAGTTTAAATAAATTTAAATTTCAAATTTTTAAATAGAATTTGAATTGACTCGTTAATATAATAAATAAATTTATTATGAATACTGTTTGAATTAAACTCAAATTTAATATTTATTTATGTCTATAAATGAAATTTAAATTTTTAATTTTATTTTTTAATAAATTTATTTTATCAAAATTTAGTTTAGATCGATTTGATTAGAGGTGAATATAAATTTTTTTTAGATTTTATGTATCTAAAATAAAGAAATAAATGATTAATAAGCACAGAAGTGAATTTTTGTAAACAAATTAATGAAATTTAAGTCCCCATGCGTTGGTGAAGGCAGGTGCAATAATTCAAACGTGCGTGCGGATATATAATAAAACAGTTAGCAATTATAACACGTGGCGCTAGAGAATCCCAACTACCTTCCGTTTTCTCTATTGTCACGAGTTACAAATGAAATCACCTACCAACTCTGTGACTATTAATTCTTTATTTTATTTACTTTACCGTTTATTATTTTACAGACGTGGTAGCTGGTGCTGTACCACCACGTGCCAATAATTTTAATGTTTAACGGAGTATTCAATATTACGTATAAAATAAATATTTATTAAAAATATTTTATTCAATTTATAATATATAAATTTATCTCAATATTTACTTTATTTATAAAAATCAAACTTAAAACAAATTCGTCAAAAGTACAGAATTTTAATTTGTTCTCATATGCAAGAAGTCTATTCTCGTAGGATGAGTTTAGTGCACGAACTTCAATGACATAGGTGTTGAGCTCCTTGTCATCGATTTGATATTACAAATTTTATTTTTTAAAAAATAAAAAATATAAAAATTATTATATAATTTTATTAAATTTTTAGTTTAAAATTTATAATTTAATATAAAACATTTAATTTAATTTTATATTTAAATAATATCTCATAACATCATATCATTCATTAATAAATTTTAATAGGCAAATTGAGTAAATTTGAAATTTATAGTGGGAGTAAATGAAAGCTTTGGCAACTTGCCTCGATGATTGAAGTTCAATAATCAGAAAAGTACGAAAAAGCCAGAAAGTCATAAGGGAAACTAGAGGAAAAATTAAATAGAATTGGCGTATACATCATTAAATTTCCACCAAACAAATTGCACAATCTTCATTTGGAATCTTATTTCTTGGCTTTAATTATATCAGCATCTTTCCTTCCTTTCTTTTAAATAAAATAAAATACATACACTCCTGCCTTTATTAGCCGAAGGTTTTGGGCTTTTCCTTTTTTTATTTTGGTATTTAGTCTGTATATAAAAATGGTATTAAAATAAAAACGTGCTCACCCAACTCTTGATATAAGTAAATTTTTAGAGTCGATTATATAAAGGGCGTGGGGGGTGCTTGCAACTCCACAAGGGCAGGAAACCAAAACAAAATTAAAG

***pDJ3S:***

GGAGAGACTTCGAGACTCCTTGATGATATCAAACTCCTCCACTTCATGTAAAAGTTCCAGCTTCTTGAGTTTAATGGAGCCAAAGGAATGTTTAGCCCAATGTCTTAAGGACCCCCGGAGACTAGCAAGTTTTTTCGCAAGCGTGAACGCCCCACATCCCACCGGCGTCGGGGTACCCCACCAGAGCGCAACGAGATCCCGAAACCCTTCCGCAGTGAGCTAAGCCAACTCGAATAGGAAGGGCCTAGGACGAGAGAGGAAAGTTCGAAGCTCGAGGCAAATGGGAACATGATCAGATCCCAAGCGGGGTAGACTTTTTTGGATAAGCCTGGGGAAAAGGTCAAGCCACTCACTATTGACAAGGAATCTATCCAACTTAACCCAGATGGGATTTGCTTGGCCATTGGTCCACGTAAACTTTCTACCTACCGAAGGTGGCTCCCCCAACCCCAGGTCCGACACAAAAGTGGTGGCATTACGGAGGTCCTCCAGGTTTGGGGTACCGGAACACTTACAACTGGGGTAGAATATCGCATTAAAATCCCCACAAACAATCCACGGGACTTCAATGCCAGAACCACAATCCCTCAGCTGATCCCAGAAGGCGCACTTCTGATCACGAGCATTAGGTCCATATACGGTAGTGCATCGCCACAAGAGATTGTCTAGTAGGTCTTATTTTTACCTCTTTATTATTTTAATTTACTCCTTAAAAATACTTAAGTGAAATTAATATTCCTAAAAATATAGATATTGCATAAATTGCTAAAGTTTTAGAAGGATTTATAGCATATATACTCACGTACACGTATATCTGGAAGATTTTTTTAAATTTTTTTATTTACTTTTTTATTTTTTTATTTTTTTAAAAGAGCTACCACTTTAGCTGGTTTATTTTTATTGAGTACAAATCCTGGACAACATGGTTACAAGAAAACAAGCAACTAAACATTTTACAAATACAAATTCAAGCTAAGTTGTATGGAACGAATGGTAAGGTCTTGTACTGAGAGAGATTTCTGTAGTGTTGGAATGCGAGCTCTGTCTGTTGTAGGGAATAATACCCTCTCTAACCTATTGGTGTGTGTGTTTTATCTACTGCCTTCTGTACGGCTCCCCATGTATGATATATGGCAACCTTGCTTTCAGAAAATAAGACAAAATATTCTGGTTTAAATACCACAAAACAAACACTACCGTAGACATTTAGAATTTCTCCGCTCTCAAAAGTTAAAAGAAAAAAAAAGTCTATTGATTCTCTTCTATCATATGTGGCTGAGAGGAGAAAAACAAAGCCTTCTAAGGCTTCATGCCTTCCTAAAATCTTTGTATCTTATTTTTTTTATTTTGTGGATTGGGATACTAATATTAATTTGTTAAATTATGAAGACAATTGCGGAAAAAAAACAATTTTTGAAGATTTTAAAGAGTTATATAATTTTATAAATAAACCTAAAATTTTCATTCAAAAACAGGAAGTGATATATATAAAGACCAATATATTGATATACATTTTGCAATTAAGTTAATACAAATTCCTTCCTATTTCTATTGTACAAGTGAATAACAAGCTACTCATTTCCCCAAACACTGCACACAGGAAAAAAAAATTATTACTCTCTTTTGTTTTCTTGTTCTCAGGTGAAAAAAAAAATGAACGAAAGAAAATATATACCGGGTCATATCTCTCTCTCTCATATATATATATCTTATGAAAAGTACTTCAAGGGTGTCTATGCTATTACTTGTCTTATCAACTTCTAAATCAACACCTACCACGTCTCCTAGCACTTGTTATACATGGTAGAGCATCTTTAATTACAAAACCACATCCCCACACACCATTAATGAAATCTATAAATTAAAGAGGGCACAACTCTAAACCCACTCCAATCTAAGCATTCGCAAGGAATTAAGAAACATAACAATC

**Fig. S1 pDJ3S:RUBY transgenic callus phenotype.**


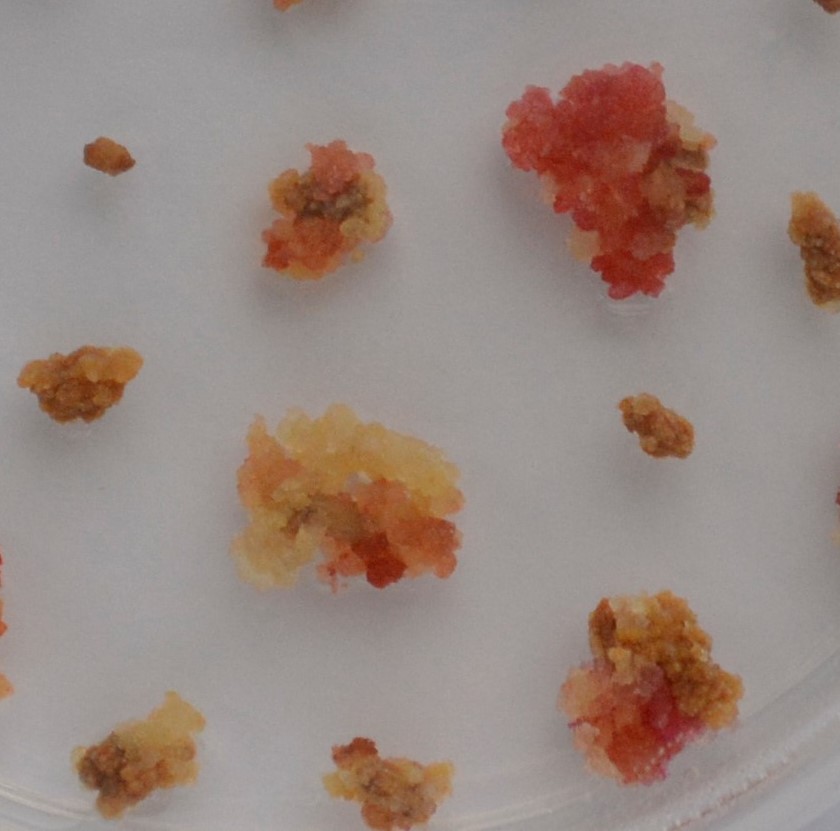


**Fig. S2 p15:RUBY transgenic callus phenotype.**


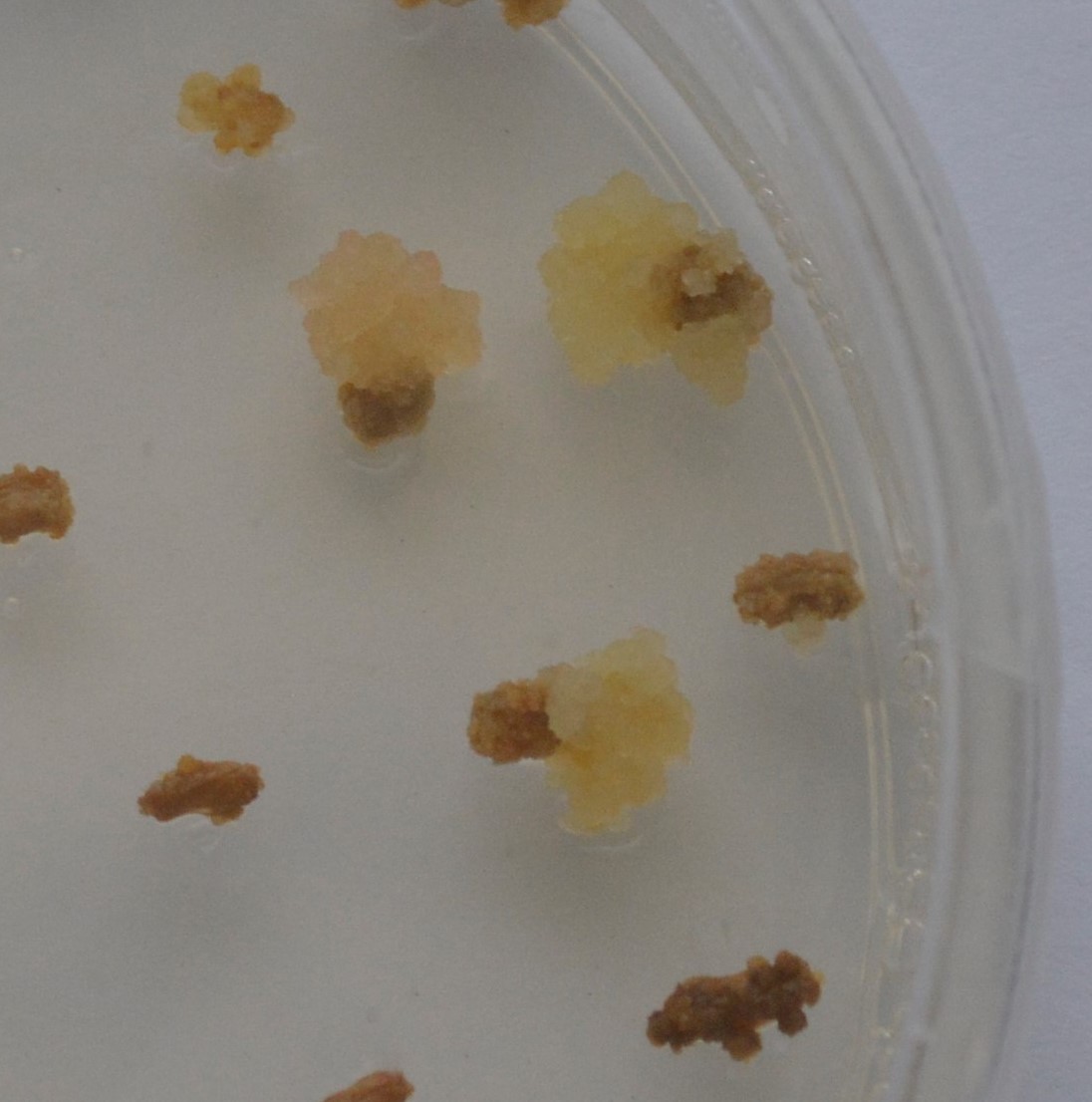

Supplement: Web_Material_uhad024 [file web_material_uhad024.docx]
